# Factors Associated with Hepatitis B Vaccination Status Among U.S. Adults with Diabetes

**DOI:** 10.3390/diseases13100324

**Published:** 2025-10-01

**Authors:** Douwné L. Müller, Jessica Yingst, William A. Calo, Heather Stuckey, Thomas Godfrey, Li Wang

**Affiliations:** 1 Department of Family and Community Medicine, Pennsylvania State University College of Medicine, Hershey, PA 17033, USA; 2 Department of Public Health Sciences, Pennsylvania State University College of Medicine, Hershey, PA 17033, USA; jyingst@pennstatehealth.psu.edu (J.Y.); wcalo@pennstatehealth.psu.edu (W.A.C.); thomasgodfrey@sbcglobal.net (T.G.); lwang1@pennstatehealth.psu.edu (L.W.); 3 Penn State Cancer Institute, Hershey, PA 17033, USA; 4 Department of Medicine, Humanities, Pennsylvania State University College of Medicine, Hershey, PA 17033, USA

**Keywords:** hepatitis B, diabetes, hepatitis B vaccination, hepatitis B virus, NHANES

## Abstract

**Background/Objectives:** Adults aged 19–59 with diabetes are recommended by the Advisory Committee on Immunization Practices (ACIP) to receive vaccination against Hepatitis B Virus (HBV) infection because of their increased risk of contracting HBV. This study aimed to examine hepatitis B (HepB) vaccination rates among U.S. adults aged 19–59 years with diabetes and explore sociodemographic factors associated with HepB vaccination. **Methods**: Data from the 2015–2018 National Health and Nutrition Examination Survey (NHANES) were analyzed to compare HepB vaccination between adults with and without diabetes. Weighted Chi-square analysis was used to test the associations between HepB vaccination status and various categorical variables. Weighted logistic regression was employed to identify factors associated with being fully vaccinated. **Results**: A total of 5988 adults aged 19–59 were included in the study, of whom 504 (8.4%) had diabetes. The HepB vaccination rate was 32.3% for those with diabetes vs. 43.6% for those without diabetes (*p* = 0.01). However, after adjusting for other covariates, having diabetes was not associated with being fully vaccinated against HBV (*p* = 0.583). Adults aged 45–59 years were less likely to be vaccinated against HBV compared to those aged 19–29 (OR: 0.12, *p* < 0.0001). Having health insurance, being female, and having a higher educational level were all associated with HepB vaccination status (all *p* < 0.01). Overall, the HBV infection rate was 1.1%. Having HepB vaccination was associated with a lower risk of HBV infection among both groups with and without diabetes. **Conclusions**: HepB vaccination among U.S. adults with diabetes was suboptimal and lower than among those without diabetes. Age and education were associated with being fully vaccinated against HBV. Future research is needed to identify and better understand barriers to receiving HepB vaccines.

## 1. Introduction

Hepatitis B virus (HBV) infection is a severe health problem that can result in considerable morbidity and mortality [1,2]. The HBV causes acute and chronic inflammation of the liver, with approximately 2–6% of acute HBV infections progressing to develop chronic HBV infections in adults [2,3]. Long-term liver inflammation leads to scarring and loss of liver function. If left untreated, it causes liver cancer and liver failure [4]. Liver failure is marked by a build-up of toxic elements in the bloodstream, significantly rising levels of bilirubin in the body, categorized by jaundice, and eventually a back-up of venous blood into the abdomen and lower extremities, ultimately leading to kidney and heart failure. Furthermore, the immune system is compromised, as is mental health. It is estimated that in the US, 862,000 to 2.2 million people live with chronic HBV. Of those, 67% will not even realize they are infected until complications set in [5,6]. In 2019, an estimated 20,700 new cases of acute HBV infection occurred, with 13,859 cases of chronic HBV infection reported [7].

There are many risk factors for HBV infection among adults. Diabetes predisposes individuals to HBV infection, likely due to a combination of impaired immune responses and repeated percutaneous exposures during routine diabetes management (glucose monitoring and insulin administration) [8,9]. In 2021, the United States (US) had 38.4 million people (11.6%) with diabetes, with 1.2 million (0.4%) newly diagnosed cases each year [10]. People with diabetes are categorized either as Type 1, usually juvenile onset and insulin-dependent, or Type 2, adult-onset, managed at the start with diet, exercise, weight loss, and later oral medication. Many with Type 2 progress to insulin dependence. In the United States, the increasing prevalence of obesity among the population has been associated with an increased incidence of diabetes. Approximately 7.6 million (29.4%) adults aged 18 years or older have a diagnosis of prediabetes, which is often reversed with weight loss [10]. Each of these groups described above is at some risk of contracting HBV. Although most outbreaks occurred in institutional settings due to the sharing of diabetes care equipment among multiple individuals, Schillie et al. [8] proved that HBV infection among non-institutionalized individuals with diabetes was also high. The results showed that the prevalence rate of HBV infection among adults with diabetes is 60% higher than among adults without diabetes [8]. Another study found that adults with diabetes (aged 23–59) have twice the risk of contracting acute HBV with no HBV risk behaviors [11]. Because of the increased risk of HBV among people with diabetes, in 2011, the Advisory Committee on Immunization Practices (ACIP) recommended that all previously unvaccinated adults aged 19 through 59 years with diabetes be vaccinated against HBV or as soon as possible after a diagnosis of diabetes is made [1].

According to a National Academy of Medicine report, HBV vaccines effectively prevent 95% of HBV infections across the entire population, with a vaccination series given as three injections over six months [6,12,13]. The HepB vaccine is a recombinant vaccine that contains Hepatitis B Surface Antigens (HBsAg) and is highly effective and safe in all ages [1,2,8]. Like other vaccines, the response rate declines with increasing age, obesity, and the presence of comorbidities [8]. Evidence also suggests that individuals with diabetes may have lower seroconversion rates compared to the general population [8], underscoring the importance of monitoring immune response and ensuring adequate protection in this high-risk group. While people with diabetes have a greater risk for HBV infection and, thus, have a greater need for vaccination, current studies suggest that overall hepatitis B (HepB) vaccination rates among all adults and those with diagnosed diabetes are low [14,15,16]. In 2018, only 30% of all adults aged 19 and over were vaccinated against HBV [17], a slight increase from 2014 and 2015, when only a quarter were fully vaccinated [6,13]. Another study in 2015 found that none of the 100 eligible participants completed the three-dose series against HBV [14]. Other studies found that overall HepB vaccination rates among those with diabetes aged 19–60 years were only 20.2% [15], while another study in 2015 reported a vaccination coverage of 24.4% for ages 19–59 [16]. These rates are slightly lower than the overall vaccination rates among US adults at high risk for HBV infection, which was 24.8% in 2015. A new report from this year found self-reported HepB vaccination rates for 2018 to be 33.0% among adults with diabetes aged 19–59 years [17]. Several studies have shown the increased risk of HBV infection and low HepB vaccination rates among adults with diabetes. We will, therefore, look at national survey data to measure the HepB vaccination rates among those with diabetes and assess factors associated with vaccination, using data years after implementing the recommended ACIP recommendation.

This study aims to calculate HepB vaccination rates and examine associated determinants among adults aged 19–59 with Type 1 or Type 2 diabetes in the National Health and Nutrition Examination Survey (NHANES) data. The results of this study could show whether the ACIP recommendation has increased vaccination among adults with diabetes, as well as identify key sociodemographic factors related to vaccination among people with diabetes. These results can be used to develop methods to improve vaccination rates among this at-risk population.

## 2. Materials and Methods

### 2.1. NHANES Data

Data for this study were analyzed in 2021 and obtained by combining two data cycles (2015–2016 and 2017–2018) of NHANES to form the analytical dataset from 2015 to 2018 [18]. NHANES is a national survey that assesses the health and nutritional status of adults and children of the US non-institutionalized population. The National Center for Health Statistics of the Centers for Disease Control and Prevention (CDC) collects data through interviews, physical examinations, and laboratory data. The survey is conducted continuously, with data being released every two years. All analyses performed in this study utilized weighted statistics. The weighting of sample data permits analysts to produce estimates of statistics that accurately represent all U.S. non-institutionalized civilians [19]. This study was not considered Human Research by the Pennsylvania State University Institutional Review Board.

### 2.2. Study Population

This study included survey participants aged 19 through 59 years in the analysis. This age range was selected to match the ACIP recommendation’s age range [1]. Eligible participants were aged 19–59 years with complete data on HBV infection, HepB vaccination, and diabetes status; individuals with missing variables of interest were excluded. A person with diabetes was defined as a person who responded “yes” to the following question: “Other than during pregnancy, have you ever been told by a doctor or health professional that you have diabetes or sugar diabetes?”

### 2.3. Outcome Measures

A person was considered to have had an HBV infection if they answered “yes” to the following question: “Has a doctor or other health professional ever told you that you have Hepatitis B? (Hepatitis is a form of liver disease. Hepatitis B is an infection of the liver from the Hepatitis B virus (HBV).” A person was considered vaccinated against HBV if they responded “yes” to the following question: “Have you ever received the 3-dose series of the hepatitis B vaccine?” For this study, if fewer than three doses of HBV vaccines were reported, the series was presumed incomplete and considered not fully vaccinated. The dataset also provided information on key sociodemographic characteristics, including age, gender, race/ethnicity, education level, country of birth, poverty income ratio (PIR), and health insurance coverage. Country of birth was defined as either being born in the U.S. or another country. A PIR below 1.3 indicated that the family is below the poverty threshold.

### 2.4. Statistical Analysis

All analysis was generated using SAS^®^ software version 9.4 (SAS Institute, Cary, NC, USA). Weighted analysis was applied to account for the complex survey design, allowing the sample to represent the US non-institutionalized population. We used the built-in SAS commands “*proc surveyfreq*” and “*proc surveylogistic*” to conduct the weighted analysis, which considered weight, stratum, and cluster information. Descriptive statistics were calculated and included sociodemographic variables (age, gender, race, education level, country of birth, poverty income ratio), health insurance coverage, and diabetes status, as well as outcome variables, including HBV infection status and HepB vaccination status. Weighted Chi-square analysis was used to test differences in categorical outcome variables between adults with and without diabetes. The weighted Chi-square test was also used to test the associations between HepB vaccination status and demographic variables and between HepB vaccination status and HBV infection among adults with and without diabetes. Lastly, multivariable logistic regression was performed to identify which independent variables were associated with the dependent variable HepB vaccination status. A two-sided significance level of <0.05 was used for all statistical tests.

## 3. Results

### 3.1. Participants’ Characteristics

A total sample of 5988 adults aged 19–59 was included in the study (Table 1). There were 504 (8.4%) adults with diabetes and 5484 (91.6%) without diabetes. The overall HBV infection rate was 1.1%, with an overall HepB vaccination rate of 42.8% among all participants (Table 1).

Among participants with diabetes (*n* = 504), the majority were aged 45–59 years (70.6%), male (52%), non-Hispanic white (53.7%), had earned a high school degree or less (44.5%), were born in the US (79.8%), had a PIR ≥ 3.5 (35.3%), and were insured (87.8%). The overall HepB vaccination rate among adults aged 19–59 with diabetes was significantly lower (32.3%) compared to those without diabetes (43.6%) (*p* = 0.01). In addition, compared to those without diabetes, the overall vaccination rate for those with diabetes was significantly different when it came to age (*p* < 0.0001), education level (*p* = 0.0003), and health insurance coverage (*p* = 0.013) (Table 1).

### 3.2. Factors Associated with HepB Vaccination

As shown in Table 2, within the diabetes group, the rate of being fully vaccinated differed by age, with the oldest age group being the least likely to be vaccinated (28.8% vaccinated for age 45–59 compared to 35.0% for age 30–44 and 65% for age 19–29; *p* = 0.024). Adults with diabetes were also more likely to be vaccinated the higher their education level. For adults without diabetes, full vaccination rates varied by age, gender, race, education level, place of birth, and health insurance coverage. Older adults, males, and Hispanics were less likely to be vaccinated, while those with higher education, born in the U.S., and with health insurance were more likely to be vaccinated.

Table 3 shows the results of the weighted logistic regression predicting HepB vaccination status among all adults and among adults with diabetes. Among those with diabetes, compared to those aged 19 to 29, adults aged 30 to 44 years (Odds ratio [OR]: 0.241; 95% Confidence Interval [CI] = 0.082−0.710; *p* = 0.012), and adults aged 45 to 59 (OR: 0.18; 95% CI = 0.053−0.580; *p* = 0.006) were less likely to be vaccinated against HBV. Also, compared to those with a high school degree or less, those with some college degree education were more likely to be vaccinated (OR: 2.45; 95% CI = 1.006−5.973; *p* = 0.049). However, the OR for college graduates or above was not statistically significant compared to those with high school or less. The ORs for gender, race, and health insurance coverage were not statistically significant. For a the model among all adults, compared to those aged 19 to 29, adults aged 30 to 44 years (Odds ratio [OR]: 0.27; 95% Confidence Interval [CI] = 0.224−0.333; *p* < 0.0001) and adults aged 45 to 59 (Odds ratio [OR]: 0.12; 95% Confidence Interval [CI] = 0.104−0.143; *p* < 0.0001) were less likely to be vaccinated against HBV. Females were more likely to be vaccinated compared to males (OR: 1.66; 95% CI = 1.395−1.968; *p* < 0.0001). Also, those with some college or college and above were more likely to be vaccinated compared to those with high school or less. Lastly, those with no health insurance were less likely than those with health insurance to be vaccinated against HBV. The ORs for race, place of birth, and PIR were not statistically significant (Table 3).

### 3.3. HepB Vaccination Status and HBV Infection

Table 4 shows the association between HepB vaccination status and HBV infection status among adults with and without diabetes, respectively. Among adults with diabetes, the rate of HBV infection was 0.3% among those fully vaccinated compared to 2.9% of those not vaccinated (*p* = 0.006). Whereas among adults without diabetes, the rate of HBV infection was 0.6% among those fully vaccinated compared to 1.3% among those not fully vaccinated (*p* = 0.014). The results showed that HepB vaccination was significantly associated with lower HBV infection and significantly protects both groups from HBV infection.

## 4. Discussion

This study used a nationally representative survey database to examine HepB vaccination rates and factors related to vaccination status among adults with diabetes. Our study found that the overall diabetes rate was 8.4%, slightly higher than prior research using NHANES data, which saw a rate of 7.4% [8]. Similar to previous work, we found that the HepB vaccination rate was lower for adults with diabetes (32.3%) than for those without diabetes (43.6%) [20,21]. While these vaccination rates remain low, they are higher than previous findings using 2009 National Health Interview Survey (NHIS) data; 16.6% among adults with diabetes and 26.5% without diabetes [21]. However, these lower HepB vaccination rates may be because the previous study was performed before the 2011 ACIP recommendation. In contrast, our study looked at HepB vaccination rates four years after the ACIP implemented the recommendation. The difference in HepB vaccination rates could also be due to more younger adults being included in the data who were vaccinated when they were children.

Though our study found higher HepB vaccination rates than those before 2011, the rates were still alarmingly low for older age groups. For instance, adults with diabetes aged 45–59 had a vaccination rate of 28.8%, whereas those aged 30–44 had a rate of 35%, compared to the 65% for those aged 19–29 years. These rates could be explained by the fact that in 1991, the HBV vaccine was recommended by ACIP for all newborn infants irrespective of diabetes status [6]. This may explain the much higher vaccination rate for the younger adult group and the lower vaccination rate for the older adult group in our 2015–2018 data. The lower rates among older adults could be explained by the HBV vaccine being first recommended in 1982 for only those considered high-risk (not including diabetes) [6,22]. Therefore, those in the age group of 30–59 years in our study were not vaccinated during their childhood. Our study also found that the overall vaccination rate of the younger adult group was only 65%, which is still much lower than one would expect for this group. Given the lower HepB vaccination among older adults, it is essential to promote HepB vaccination among older age groups. This may be accomplished by educating providers to target the older age group and by obtaining health insurance companies to encourage and incentivize older clients to obtain the vaccine.

Our study has also identified several factors associated with low HepB vaccination rates. Our results showed that age and education level influence HepB vaccination status, similar to previous research using national datasets (NHIS and NHANES) [15]. Both our study and previous research showed that as age increases, the odds of being vaccinated decrease, and that having some college education was associated with being vaccinated [15,20,23]. However, these previous studies have also shown other factors that affect HepB vaccination, such as foreign birth and Hispanic ethnicity [15], being a healthcare provider, having been tested for HIV, having ten or more healthcare visits in the previous year [20], as well as being male, insured, and having chronic liver disease [23].

Regarding the HBV infection, our study only performed a univariate association test with the HepB vaccination status, without considering other variables due to data availability. It should be noted that our study focused only on sociodemographic variables and health insurance coverage. This meant that other known risk factors of HBV infection were not investigated. Future research could consider additional variables such as access to care variables and variables related to HBV infection risks, such as IV drug use, sex between men, number of sex partners, HIV status, healthcare workers, persons with chronic liver disease, and persons with kidney disease [15,20,24,25].

Low HepB vaccination rates can be due to the lack of Electronic Medical Record (EMR) alerts, which remind providers that the adult with diabetes should be receiving the recommended HBV vaccine, or that the follow-up vaccine is needed. Hechter et al. [26] discovered that using EMR reminders to increase HBV vaccine initiation and series completion rates among adults with diabetes was highly effective. Therefore, changes within the healthcare system and primary care practice are needed when managing care for adults with diabetes. These changes can include HepB vaccination as part of the standard of care for those with diabetes and implementing EMR alerts/reminders in their system.

Another reason for the low HepB vaccination rate could be that many individuals manage their diabetes through appropriate medication, continued glucose monitoring, diet, and exercise, and may not necessarily be at an increased risk for HBV. Because they do not manage their diabetes with percutaneous glucose monitoring or insulin injection, physicians or adults with diabetes may not consider them or themselves to be high-risk individuals. Future research is, therefore, needed to study whether all people with diabetes are at an increased risk for HBV and whether the type of diabetes and the way adults with diabetes manage their diabetes should be considered.

Our study also showed evidence that the HepB vaccination may be beneficial in preventing infection. We found the association between HepB vaccination and HBV infection statistically significant among adults with and without diabetes. The rate of HBV infection was lower when fully vaccinated (0.3% for those with diabetes and 0.6% for those without diabetes) compared to those not fully vaccinated (2.9% and 1.3%). Our univariate analysis results echo previous research on newly infected individuals and found that those with diabetes were less likely to have been vaccinated for HBV than those without diabetes [25].

### Limitations

This study also had several limitations. First, participants with missing data were excluded from the analysis without imputation. The missingness was likely to be random and would not cause much bias. Secondly, the association between HepB vaccination status and HBV infection needs to be interpreted with caution, for it is unknown whether HBV infection occurred before or after HepB vaccination. Future research can use EMR data to overcome this issue. Thirdly, since HBV infection in this study was defined based on self-reported physician diagnosis, this may lead to an underestimation of HBV infections. Lastly, other HBV risk factors were not included in this study, which may change the observed correlations between HepB vaccination and HBV infection. Therefore, the effect of HepB vaccination may be either underestimated or overstated due to unmeasured confounding factors.

## 5. Conclusions

Overall, our findings showed that HepB vaccination rates among adults with diabetes were suboptimal. The older age groups were less likely to be vaccinated against HBV, which highlights the importance of increasing HepB vaccination among older adults. We also found that among adults aged 19–29, who should have been vaccinated in infancy, the vaccination rate was only 65%, raising concern about gaps in vaccine coverage and potential vulnerability to HBV infection. Awareness of the ACIP recommendation among providers and patients is needed to increase vaccine coverage for adults with diabetes. Healthcare systems and primary care practices should also consider including HepB vaccination for adults with diabetes as part of their standard of care. Future research is needed to better understand the barriers adults with diabetes have in receiving the HBV vaccine and whether the recommendation for adults with diabetes is being implemented.

## Figures and Tables

**Table 1:** Characteristics of adults aged 19–59 years with and without diabetes.

	All Adults		Persons with Diabetes		Persons Without Diabetes		
Characteristics	Sample (*n*)	Weighted %	Sample (*n*)	Weighted %	Sample (*n*)	Weighted %	*p*-Value
**Total**	5988	-	504	8.4	5484	91.6	
**Age (years)**							<0.0001 ***
19–29	1487	24.2	24	5.2	1463	25.7	
30–44	2191	35.8	117	24.2	2074	36.7	
45–59	2310	40	363	70.6	1947	37.6	
**Gender**							0.396
Male	2813	48.5	254	52.0	2559	48.2	
Female	3175	51.5	250	48.0	2925	51.8	
**Race/Ethnicity**							0.081
Hispanic	1568	17.0	152	19.6	1416	16.8	
Non-Hispanic White	1822	59.7	126	53.7	1696	60.1	
Non-Hispanic Black	1400	12.8	135	15.7	1265	12.6	
Other	1198	10.5	91	11.0	1107	10.5	
**Education**							0.0003 **
High school or less	2535	36.0	248	44.5	2287	35.4	
Some College	1926	32.5	171	35.1	1755	32.3	
College Graduate or above	1527	31.5	85	20.5	1442	32.3	
**Place of Birth**							0.815
United States	4031	80.3	334	79.8	3697	80.4	
Other	1957	19.7	170	20.2	1787	19.6	
**Poverty Income Ratio**							0.295
≤1.3 (low)	2313	28.6	206	32.1	2107	28.4	
>1.3 to <3.5	2058	31.7	177	32.6	1881	31.6	
≥3.5 (High)	1617	39.7	121	35.3	1496	40.0	
**Health Insurance**							0.013 *
Yes	4732	83.4	417	87.8	4315	83.0	
No	1256	16.6	87	12.2	1169	17.0	
**Fully Vaccinated**							0.01 *
Yes	2487	42.8	157	32.3	2330	43.6	
No	3501	57.2	347	67.7	3154	56.4	
**HBV Infection**							0.15
Yes	75	1.1	8	2.0	67	1.0	
No	5913	98.9	496	98.0	5417	99.0	

Boldface indicates statistical significance (* *p* < 0.05, ** *p* < 0.01, *** *p* < 0.001).

**Table 2:** Weighted Chi-square test of the association between HepB vaccination status and demographic variables.

	*Diabetes*			*Without Diabetes*		
	Fully Vaccinated *n* (%)	Not Fully Vaccinated *n* (%)	*p*-Value	Fully Vaccinated *n* (%)	Not Fully Vaccinated *n* (%)	*p*-Value
**Overall**	157 (32.3)	347 (67.7)		2330 (43.6)	3154 (56.4)	
**Characteristics**						
**Age (years)**			0.024 *			<0.0001 ***
19–29	16 (65.0)	8 (35.0.)		969 (69.4)	494 (30.6)	
30–44	45 (35.0)	72 (64.4)		872 (43.4)	1202 (56.6)	
45–59	96 (28.8)	267 (71.2)		489 (26.2)	1438 (73.8)	
**Gender**			0.114			<0.0001 ***
Male	62 (27.9)	192 (72.1)		903 (37.0)	1656 (63.0)	
Female	95 (37.1)	155 (62.9)		1427 (49.7)	1498 (50.2)	
**Race/Ethnicity**			0.474			0.005 **
Hispanic	38 (25.9)	114 (74.1)		505 (37.5)	911 (62.5)	
Non-Hispanic White	43 (34.7)	83 (65.3)		753 (45.2)	943 (54.8)	
Non-Hispanic Black	41 (32.3)	94 (67.7)		507 (40.6)	758 (59.3)	
Other	35 (32.0)	56 (68.0)		565 (48.0)	542 (52.0)	
**Education**			0.026 *			<0.0001 ***
High school or less	50 (22.7)	198 (77.3)		696 (31.7)	1591 (68.3)	
Some College	71 (42.6)	100 (57.4)		851 (48.3)	904 (51.7)	
College Graduate or above	36 (35.6)	49 (64.4)		783 (51.9)	659 (48.1)	
**Place of Birth**			0.420			<0.0001 ***
United States	113 (33.3)	221 (66.7)		1653 (45.3)	2044 (54.7)	
Other	44 (28.5)	126 (71.5)		677 (36.6)	1110 (63.4)	
**Poverty Income Ratio**			0.262			0.548
≤1.3 (low)	54 (27.7)	152 (72.3)		830 (41.7)	1277 (58.3)	
>1.3 to <3.5	58 (30.5)	119 (69.5)		807 (44.0)	1074 (56.0)	
≥3.5 (High)	45 (38.1)	76 (61.9)		693 (44.6)	803 (55.4)	
**Health Insurance**			0.347			<0.0001 *
Yes	138 (33.2)	279 (66.8)		1964 (45.7)	2351 (54.3)	
No	19 (25.8)	68 (74.2)		366 (23.3)	803 (66.7)	

Boldface indicates statistical significance (* *p* < 0.05, ** *p* < 0.01, *** *p* < 0.001).

**Table 3:** Weighted multivariable logistic regression of HepB vaccination (3 doses).

	*3 Doses of the HBV Vaccine*					
	*All Adults*			*Diabetes*		
** Characteristics **	**OR**	**95% CI**	***p*-Value**	**OR**	**95% CI**	***p*-Value**
**Diabetes** (Ref: Yes)						
No	0.89	0.591–1.352	0.583	-	-	-
**Age (years)** (Ref: 19–29)						
30–44	0.27	0.224–0.333	<0.0001 ***	0.24	0.082–0.710	0.012 *
45–59	0.12	0.104–0.143	<0.0001 ***	0.18	0.053–0.580	0.006 **
**Gender** (Ref: Male)						
Female	1.66	1.395–1.968	<0.0001 ***	1.53	0.828–2.823	0.168
**Race/Ethnicity** (Ref: Hispanic)						
Non-Hispanic White	1.18	0.926–1.508	0.171	1.28	0.713–2.288	0.397
Non-Hispanic Black	0.92	0.712–1.184	0.498	1.11	0.583–2.107	0.746
Other	1.27	0.955–1.675	0.098	1.31	0.645–2.647	0.445
**Education** (Ref: High school or less)						
Some College	2.11	1.766–2.526	<0.0001 *	2.45	1.006–5.973	0.049 *
College Graduate or above	2.51	1.924–3.267	<0.0001 *	1.68	0.559–5.044	0.343
**Place of Birth** (Ref: United States)						
Other	0.85	0.701–1.027	0.089	1.10	0.598–2.035	0.747
** Poverty Income Ratio** (Ref: ≤1.3 (Low))						
>1.3 to <3.5	0.96	0.741–1.234	0.721	0.99	0.509–1.913	0.968
≥3.5 (High)	0.99	0.761–1.297	0.961	1.23	0.552–2.736	0.603
** Health Insurance ** (Ref: Yes)						
No	0.66	0.524–0.828	0.0008 *	0.67	0.323–1.378	0.263

Boldface indicates statistical significance (* *p* < 0.05, ** *p* < 0.01, *** *p* < 0.001). OR, Odds Ratio; CI, Confidence Interval.

**Table 4:** Weighted Chi-square test of the association between HepB vaccination status and HBV infection status.

	Diabetes (p = 0.006 **)		Without Diabetes (p = 0.014 *)	
	HBV Infection	No HBV Infection	HBV Infection	No HBV Infection
**Vaccinated**	1 (0.3)	156 (99.7)	20 (0.6)	2310 (99.4)
**Not Vaccinated**	7 (2.9)	340 (97.1)	47 (1.3)	5417(98.7)

Boldface indicates statistical significance (* *p* < 0.05, ** *p* < 0.01).

## Data Availability

The original data presented in the study are openly available in the National Health and Nutrition Examination Survey (NHANES) repository, maintained by the National Center for Health Statistics (NCHS) at the Centers for Disease Control and Prevention (CDC), and are publicly accessible at https://wwwn.cdc.gov/nchs/nhanes/Default.aspx (accessed on 10 September 2025). The specific NHANES cycles and variables used are detailed in the Methods section.

## Abbreviations

The following abbreviations are used in this manuscript:

ACIP	Advisory Committee on Immunization Practices
HBV	Hepatitis B Virus
HepB	Hepatitis B
NHANES	National Health and Nutrition Examination Survey
NHIS	National Health Interview Survey
CDC	Centers for Disease Control and Prevention
PIR	Poverty Income Ratio
OR	Odds Ratio
CI	Confidence Interval
EMR	Electronic Medical Record
US	United States
